# Prevention of Obesity and Metabolic Syndrome in Children

**DOI:** 10.3389/fendo.2019.00669

**Published:** 2019-10-01

**Authors:** John W. Gregory

**Affiliations:** Division of Population Medicine, School of Medicine, Cardiff University, Cardiff, United Kingdom

**Keywords:** overweight, obesity, metabolic syndrome, prevention, diet, activity, environment

## Abstract

In recent decades, the prevalence of overweight and obesity has become increasingly common such that it is now the major nutritional problem worldwide. Obesity occurs when dietary energy intake exceeds energy expenditure and has arisen in many societies due to an increasingly “obesogenic” environment in which physical activity has declined and yet children continue to be exposed to unhealthy, energy-dense diets. Additional risks for the development of obesity also include psychological issues and genetic factors. Obesity has many adverse health consequences including development of insulin resistance, Type 2 diabetes, and the metabolic syndrome. There are also important genetic influences on the likelihood of developing insulin resistance. Given the limited success of therapeutic interventions to treat obesity and the metabolic syndrome, there has been an increased interest in preventative strategies. These are likely to be most successful when targeting the young and will require a combination of approaches which will need inter-disciplinary collaborations across health and local government to target families, schools, and local environments to facilitate behavior changes which influence young people's eating behaviors and habitual levels of physical activity.

## Introduction

Overweight and obesity are significant risk factors for developing the metabolic syndrome and have in recent years, become the major world-wide nutritional challenge (1), affecting both children (2), and adults in countries with both high and low incomes. Obesity arises primarily from the need for energy storage when dietary energy intake exceeds energy expenditure. The latter largely consists of basal metabolic rate which forms ~60–75% of total energy expenditure. The energy expended through physical activity which in children is usually a larger proportion (20–30%) of total daily energy expenditure than in adults, is the next largest component with energy expended in thermogenesis accounting for only about 10% (3).

The rapid increase in obesity rates worldwide has been caused by the combination of less active lifestyles and a failure to reduce energy intake in line with reduced total energy expenditure arising from reduced physical activity. Since 1980, the prevalence of obesity has more than doubled in over 70 countries with the recent rate of increase seemingly more rapid in children (1, 4). The prevalence of childhood obesity has increased 8-fold since 1975 (4) and the combined prevalence of overweight and obesity may be as high as 23% worldwide. The prevalence of obesity increases significantly after adolescence (1). Furthermore, most obese children remain obese as adults and have a 5-fold increased risk of obesity in adult life than that seen in normal weight children (5, 6).

### Consequences of Obesity

Increasing prevalences of overweight and obesity have been associated with a range of adverse health consequences including metabolic phenomena (e.g., insulin resistance, impaired glucose tolerance, Type 2 diabetes (T2D), hyperlipidaemia, steatohepatitis), endocrine disorders (e.g., advanced pubertal development, polycystic ovarian disease), cardiovascular disease (e.g., hypertension), respiratory symptoms including breathlessness and obstructive sleep apnoea, skeletal effects such as leg deformities and slipped capital femoral epiphyses, malignancies, and psychological problems such as reduced self-image, bullying and behavioral problems. Furthermore, there is evidence that overweight individuals experience reduced education and income (7) which are themselves, also risk factors for future obesity. Obesity is associated with increased mortality in adult life and premature death.

#### Metabolic Syndrome

The metabolic syndrome is a cluster of cardiometabolic abnormalities which together represent added risk factors for cardiovascular disease and T2D (8). The syndrome occurs more commonly in overweight individuals, affecting relatively few (3–4%) young people of normal weight but depending on the definition used, 26–50% of obese children and adolescents (9). It is therefore important to recognize that not all obese young people develop the metabolic syndrome though up to 90% of obese children and adolescents will have at least one component (10). Nonetheless, a significant minority will remain metabolically healthy. By contrast with white Caucasians, individuals of Hispanic, Middle-Eastern, Asian or Afro-American ethnic origin are at particular risk of metabolic complications associated with obesity (11–13).

Developing a definition of the metabolic syndrome in children has proved challenging, as adult criteria cannot be simply imposed on children, due to the variation with age, puberty, and ethnicity in a number of the variables used and differences compared to adults in their associations with morbidity. A pediatric definition of metabolic syndrome has evolved since one of the first definitions in 2003 by Cook et al. (10). A more recent definition by the International Diabetes Federation (14) is widely recommended (9) in which young people are subdivided by age. In this definition, in those <10 years, it is suggested that the metabolic syndrome cannot be diagnosed. The definitions for those aged 10–16 years and over 16 years are shown in Table 1.

**Table 1:** International diabetes federation definition of metabolic syndrome in childhood.

**Age (years)**	**Waist circumference (obesity)**		**Triglycerides**	**HDL-C**	**Blood pressure**	**Glucose**
10–16	≥90th percentile	Plus 2 of the following	≥1.7 mmol/L	<1.03 mmol/L	Systolic ≥130 mmHgDiastolic ≥85 mmHg	≥5.6 mmol/L
>16	≥94 cm (men) ≥80 cm (women)	Plus 2 of the following	≥1.7 mmol/L	<1.03 mmol/L (men) <1.03 mmol/L (women)	Systolic ≥130 mmHg Diastolic ≥85 mmHg or previously diagnosed hypertensive	Impaired fasting glucose or T2D

However, it should be recognized that given the challenges in defining metabolic syndrome in childhood [more than 40 definitions have been proposed (15)], it has been suggested that it is more important to focus attention on children with clustering of these cardiometabolic risk factors than worrying whether such children have a defined variant of the metabolic syndrome (16).

Screening for the presence of the metabolic syndrome in at risk overweight and obese young people requires a careful clinical history and examination, seeking evidence of comorbidities (e.g., symptoms of diabetes or the presence of acanthosis nigricans which is indicative of insulin resistance). The American Diabetes Association has advised an oral glucose tolerance test as screening for T2D, every 3 years from the age of 10 years or from the start of puberty in the context of the presence of any two of the following features: a family history of T2D, high risk ethnicity, signs of insulin resistance, or associated diseases (hypertension, dyslipidaemia, polycystic ovarian disease, or being born small for gestational age) or maternal history of diabetes or gestational diabetes affecting the overweight child in question (17). Blood pressure should be monitored at all healthcare contacts from the age of 3 years (18). Lipids and HDL lipid profile should be measured in overweight children between the ages of 2–8 years and repeated between 12 and 16 years old (18). Liver function testing (serum alanine and aspartate aminotransferases) is advised biannually starting from 10 years of age in obese children or those overweight with other risk factors (17, 19).

## Risk Factors for Obesity and the Metabolic Syndrome

### Genetic Influences

There is good evidence for multiple genetic mechanisms contributing to obesity. Parental body mass index is an important predictor of childhood obesity (20) and family studies suggest that the heritability of obesity phenotypes ranges from about 30–50% (20). Furthermore, there is evidence that genetic influences play significant roles in determining body fat content, energy intake, and energy expenditure. Although obesity seems a largely polygenic determinant, a number of single gene mutations (e.g., of the melanocortin 4 receptor gene, fat mass, and obesity-associated gene, leptin, and leptin receptor genes or the proopiomelanocortin gene) have been discovered which together may account for ~1% of cases of human obesity (21).

Absolute basal metabolic rate and total energy expenditure is usually increased in overweight individuals (22) due to the relatively increased amounts of lean tissue found in overweight people and the increased physical effort required to move their increased weight. However, some detailed metabolic studies have demonstrated slight reductions in thermogenesis in individuals in families with a tendency to overweight, suggesting a possible genetic basis to their predisposition to gain excess weight (23).

Obesity is associated with increasing insulin resistance which causes increased hepatic glucose production and reduced glucose uptake in muscle and adipose tissue. At the same time, evolving β-cell dysfunction arises which prevents a compensatory increase in insulin secretion. The combination of insulin resistance and a loss of compensatory insulin response leads to the development of T2D. The incidence of T2D has changed in parallel to the changes in obesity with some clinics now reporting up to 45% of all new cases of diabetes in children and adolescents being due to T2D (24). There is evidence that there is a genetic predisposition to developing insulin resistance with particularly high risks apparent in the Middle-Eastern and Asian (especially Chinese and Indian) populations who develop T2D at a lower body mass index and younger age than seen in Western populations (11). More detailed genetic studies have now shown that variants in at least 13 genes are associated with significant variations in insulin resistance (25).

It seems these are also genetic influences on how diet may impact the development of the metabolic syndrome with evidence from Iranian studies in adults that polymorphic variants of the APOC3, APOA1, MC4R, and cyclin D2 genes may all play a role (26–29).

### Early Risk Factors

A comprehensive review of early risk factors for later childhood obesity has just been published (30). This review highlights evidence that early nutritional and environmental factors play an important role on the risk of developing later childhood obesity. In particular, maternal (31) and paternal (32) obesity at conception as well as rapid gestational weight gain (33, 34) represent particular risk factors, though the failure to find a consistently stronger maternal than paternal influence on offspring adiposity casts some doubt on the fetal overnutrition hypothesis (32).

It has been recognized that the *in utero* environment represents an extra risk for developing the metabolic syndrome in later life. Insulin resistance may arise from an adverse *in utero* environment associated with intrauterine growth restriction. The Barker hypothesis otherwise known as the thrifty phenotype hypothesis proposed that an adverse *in utero* environment leading to intrauterine growth retardation and reduced birthweight are risk factors for the development of cardiovascular disease and T2D in later adult life (35).

The evidence around the protective effect of breast-feeding in preventing obesity in later life is not clear-cut. Formula-fed babies are known to gain weight more rapidly than breast-fed infants (36). The majority of meta-analyses suggest that breast-feeding is protective against developing obesity in later life (37–40), though a large study does not suggest that increased duration and exclusivity reduces obesity risk in adolescents (41). A study examining the association between duration of breast-feeding and early childhood cardiometabolic risk factors suggests a small positive benefit of longer breast-feeding albeit no extra benefit beyond 24 months (42). A high infant protein intake after weaning, also represents an additional dietary risk factor for later obesity (43).

### Diet

The current “epidemic” of obesity is thought to have arisen primarily as a consequence of reduced levels of physical activity with urbanization and an increasingly sedentary lifestyle. This has occurred without an adequately concomitant reduction in dietary energy intake partly as a consequence of increasingly energy-dense diets. Interestingly however, data on the association of fat intake with obesity have shown conflicting results with fat intake reported to have fallen during the time period that obesity has increased (44, 45). Fat content such as trans fat may play a more important role than the total amount of fat consumed (46).

The increasing rates of obesity in recent decades seem to match the increased consumption of fast foods and sugar-sweetened drinks. Studies have shown that eating fast foods more than twice weekly (47) or increased intake of sugar-sweetened drinks (48) are associated with increased body mass indices. In recent years, because of concerns that increased intake of sugar-sweetened drinks is linked to obesity, there has been a dramatic increase in the intake of non-nutritive artificial sweeteners by children (49). Some have suggested that these sweeteners also represent a similar risk to future health imposed by sugar-sweetening (50). However, a recent meta-analysis of nine cohort studies shows that although consumption of non-nutritive sweeteners is associated with an increase in body mass indices, there is thus far, no evidence of an association with other components of metabolic disease (49).

A recent review has examined dietary factors associated with development of the metabolic syndrome. This review reports a reduced risk of the metabolic syndrome arising from a diet rich in whole grains, legumes, and nuts whereas the risk is increased by a diet rich in white rice and salty or sweet snacks (29). The same group have been able to develop a modification of the US diet quality index (51) known as The Modified Healthy Eating Index and shown that this is associated with components of the metabolic syndrome in young people (52). A Dietary Approaches to Stop Hypertension (DASH) diet which emphasizes vegetables, fruits, and low-fat dairy foods with moderate amounts of whole grains, fish, poultry, and nuts and encourages reduced sodium in the diet with foods rich in nutrients that help lower blood pressure, such as potassium, calcium and magnesium has been shown in Iranian children to be associated with improved high blood pressure, high fasting glucose, and abdominal obesity (53).

The patterns of eating behavior may play an important role in predisposing to obesity. The number, duration and regularity of meals may influence risks of obesity (45). A tendency to eat in the absence of hunger leads to a >4-fold risk of being overweight (54). Missing breakfast has been associated with an increased adverse metabolic risk (55) and there is evidence to suggest that consumption of a high quality breakfast with low energy density is associated with a number of improved markers of cardiometabolic health (serum uric acid, cholesterol, and measures on insulin resistance) in a cohort of 8–12 year olds who were overweight or obese (56).

### Environment

Obesity has strong underlying cultural and socioeconomic predispositions. It is more prevalent in countries with higher incomes (1) although within countries, a reverse relationship is reported with rates seemingly higher in families of lower socioeconomic classes (57).

A child's food intake may be affected by a range of environmental influences operating within their family, school, or the wider environment. Obesity is more likely to arise in children being reared in single parent households (58) and the family approaches to food type, eating out, and habitual levels of physical activity can impact a child's rate of weight gain (45, 46). Family factors may explain up to 27% of the variance in a child's weight (58). The use of food as a reward system for young children may promote an unhealthy relationship to food (59).

The school environment may also have some limited influences on a child's eating behaviors through the presence of fast food and soft drink vending machines (60). Furthermore, peer influences have been shown within primary schools to modify children's physical activity behavior (61). Transport to and from school has changed in recent decades from walking and cycling, to being given lifts in parent's cars or taking buses (62).

The wider environment may also impact children's energy balance through the availability of safe spaces to play, walk, and cycle (63). Television and computer use may impact sedentary behavior and mobile phone or tablet use reduces the time for physical activity (64). Several studies have shown a clear relationship between low levels of physical activity (65) or increased sedentary time spent watching television, playing video games or using a computer (66, 67) and obesity. Accessibility to fast food outlets, the nutritional quality of their food and portion sizes are all important modifiers of behavior. Children are also exposed to large amounts of food and beverage advertising on television. Much of the advertising is for foods of questionable nutritional quality. Studies have shown that children's behavior, particularly in those who are overweight is influenced by such advertising, resulting in preferences for the advertised food in both the short and long term (68).

Shortened sleep duration is also associated with obesity (69) through mechanisms that are not entirely clear but may include tiredness adversely impacting physical activity or longer periods of wakefulness providing more opportunity to eat.

### Psychological Influences

Stress has been shown to impact obesity through its effects on eating behaviors, such as irregular meal times, leading to consumption of fast foods and snacks, increased eating speeds, and food amounts (45, 70). Hemmingsson has proposed a causal model which suggests that psychological and emotional distress arising from adverse experiences in childhood is the fundamental link between socioeconomic disadvantage and weight gain (70). It has been suggested that lack of resilience leads to maladaptive coping strategies such as eating to suppress negative emotions, chronic stress, appetite up-regulation, low-grade inflammation and possibly reduced basal metabolism, all of which may predispose to weight gain.

A range of adverse mental health conditions including depression, anxiety, low self-esteem, and eating disorders have been found to be more common in young people who are obese. However, whether these problems are causally related to the obesity is less clear as studies have suggested their onset to arise after the development of obesity (71).

## Prevention of Obesity

The previous section has highlighted a range of variables which are thought to be risk factors for developing obesity and the metabolic syndrome. Many of these with the exception of genetic influences are potentially modifiable targets for preventative initiatives. Given the major societal and health costs arising from obesity, it has been suggested that governments should take action (72). The World Health Organization has published a report in which it advises health policy makers around the world to consider a combination of approaches; promoting an intake of healthy food, promoting physical activity, targeting preconception, pregnancy care and early childhood diet, with physical activity and a focus on health, nutrition, and physical activity for school age children (73). In comparison, the US-based Centers for Disease Control and Prevention recommends a range of separate community strategies to prevent obesity, including promoting breastfeeding, access to affordable healthy food and beverages, promoting healthy food and beverage choices, and fostering physical activity among children (74).

It has been proposed that prevention of obesity needs an integrated approach to public health (75), developed and implemented by networks of local government, public, and private stakeholders and health promoters. Such an approach should combine multicomponent approaches that target eating behaviors (energy intake) and physical activities which contribute to energy expenditure. Secondly, multi-level approaches which target individual children, their families, primary care, and community initiatives may be necessary. Finally, a range of settings should be considered which may include the family home, schools or community facilities (75, 76). However, one of the reasons quoted for integrated approaches such as these being slow to “take-off” is the lack of evidence for their effectiveness, lack of awareness of the problem outside health, and flaws in the governmental approaches to develop relevant policies.

To try and resolve the lack of effective action, it has been suggested that the obesity epidemic be seen as a societal rather than individual challenge (77) on the basis that the problem is not taken seriously outside of the health sector, there is lack of recognition that obesity prevention is better than individually-based interventions of limited efficacy to treat obesity and the role to be played by the physical and built environments. It is suggested that governments can play a role by “nudging” health behavior, for example, designing stairs with prompts to use them or making fruit available for lunches in schools (75).

### Prevention of Obesity Risk in Early Life

There is limited evidence to suggest the effectiveness of interventions in early life to prevent later obesity. However, given the likely risk factors outlined above, it seems reasonable to promote healthy nutrition and normal weight status at reproductive age in adults contemplating having a family (30) and during pregnancy. Careful monitoring of infant growth to detect the early development of obesity would seem important. Given the evidence of widespread benefits which arise from breast-feeding, promotion of exclusive breast-feeding for the first four to 6 months of life would seem worthwhile as there is limited evidence to suggest likely protection against obesity, by reducing fat mass and impaired glucose tolerance in later life (38, 78). After weaning, infants should be exposed to a diverse diet (30) and from the age of 1 year, infants should feed with the rest of the family.

There are currently few studies which report the outcomes of initiatives targeting the prevention of obesity in pre-school-aged children (79, 80). Those that do, show improvements in body mass indices greater than those seen in interventions targeting older children. Most studies are US-based, often targeting the disadvantaged, delivered in a range of settings and impacting a range of targets including diet, promotion of physical activity, and reduction of sedentary activity so drawing broad conclusions is challenging. The consensus is that preventative measures in early childhood offer the possibility of long term transfer of knowledge and behavior patterns and that likely enhanced motor skills and reduced media consumption will be effective (81).

### Preventing Obesity in School-Aged Children

So, what is the evidence for the effectiveness of interventions to prevent obesity in older children? A recent meta-analysis highlights the discrepancies between observational and interventional studies (82). Another meta-analysis (81) reports that most interventions have been school-based and of limited effectiveness. A combination of enhanced physical activity and improved nutrition seem most likely to be beneficial in preventing obesity (82). In older children, a recent Cochrane Intervention Review reports that dietary interventions alone seem to have little effect whereas interventions focussing on enhancing physical activity alone may reduce obesity but supports the view that combined activity and dietary interventions may be effective (83). Results have generally been more impressive in the younger age groups in whom parents and teachers need to be included. By contrast, interventions in adolescents need to target the young person directly. Behavior-based interventions seem to have been of limited value and a combination of aiming for 1 h minimum of physical activity in school, promoting healthy food choices by taxing unhealthy foods and mandatory standards for meals in kindergartens and schools combined with restrictions on food advertising targeting children are advised. The diet should be varied with plenty of water or unsweetened drinks, a focus on plant-based foods but limited animal products and very little added sugar or sweets with the avoidance of snacks (81).

In the absence of robust evidence, a number of international guidelines have advocated a range of measures to reduce the risks of developing obesity (73, 84–86). These include promotion of moderate to intensive physical activity by encouraging a minimum of 20 min but preferably more activity (about one to one and a half hours and equivalent to at least 10–12,000 steps daily (87, 88). These targets should be achieved at least 5 days weekly as a core aim. Promotion of active methods such as cycling for travel to school are advised. At the same time, interventions that reduce screen (television, computer, or smart phone) time may be effective ways of targeting sedentary behavior; screen time should be reduced to a maximum of 2 h daily. The US Preventative Services Task Force recommends behavioral interventions to reduce sedentary screen time among children 13 years and younger (89) but found insufficient evidence to recommend school-based obesity programs to prevent or reduce overweight and obesity among children and adolescents (73, 85, 90).

In school-aged children, a meta-analysis (91) of 398 potentially relevant articles, identified 18 studies involving 18,141 children which met the inclusion criteria and showed no benefits on body mass indices arising from physical activity interventions, although there were other beneficial health effects. By contrast two other meta-analyses of interventions in a school-setting, suggested modest benefits on body mass indices, particularly of interventions combining physical activity with optimized eating habits; girls seemed to benefit more than boys (92, 93).

Meta-analyses of interventions in adolescents are also hampered by the heterogeneity of interventions (81). Multi-component interventions seem to produce modest beneficial effects on measures of body composition (94) but a range of study limitations prevents robust conclusions being drawn as to a favored intervention for this age-group. Again, girls seemed to benefit more than boys.

A longitudinal study on nearly 500 children over a 4 year period, has shown that the risks of childhood obesity were determined by the length of the street the child lived on, accessibility by foot of the local playground, frequency of buses, and socioeconomic status of the area (95). However, change in body mass indices was more impacted by familial/social factors, than the neighborhood environment. Another study suggested however, that only 2% of the variance in body mass indices could be explained by similar characteristics of the residential environment (96). These findings suggest that there should be caution about the potential benefit of targeted changes on the environment.

## Preventing the Metabolic Syndrome

Whereas, there are plenty of observational and interventional studies which aim to reduce the risks of childhood obesity and therefore indirectly, the metabolic syndrome, data from studies which target the metabolic syndrome specifically are rather less prevalent and often focussed on children and teenagers with pre-existing obesity. The Barker hypothesis raises the intriguing suggestion, that prenatal interventions that address maternal poverty and nutritional needs, to optimize *in utero* growth, may produce beneficial reductions in the future risk of the metabolic syndrome in later life. However, the benefits of such initiatives have yet to be shown in human studies.

However, a series of relevant papers have been published by the Tehran Lipid and Glucose Study which targets a population at high risk of the metabolic syndrome (97). This is a community-based programme, promoting a healthy life-style by targeting healthy dietary patterns and increasing physical activity, to reduce non-communicable disease risk factors including the metabolic syndrome [three of five cardiometabolic risk factors (10)]. The second phase of this study has tested a number of interventions on targeted sub-groups of the population including schoolchildren (97). A recent publication has shown that a healthy lifestyle education can reduce the short term (6 year) risk of the metabolic syndrome in children (98) with similar looking but potentially longer lasting benefits in adolescents (99).

Others have also reported the effectiveness of dietary interventions. Despite the evidence to suggest an association between increased duration of breast-feeding and subsequent cardiometabolic risk (42), an intervention designed to increase the duration of breast-feeding had no impact on blood pressure or biochemical metabolic measures at 11.5 years of age (100). By contrast, a randomized controlled trial developed to assess the impact of a DASH diet on metabolic outcomes in a group of adolescent girls, showed that 6 weeks of a DASH diet improved high blood pressure and markers for the metabolic syndrome (101). Also, there is evidence from a randomized controlled trial of breakfasts of either low or high glycaemic indices that metabolic benefits may arise from breakfasts of low glycaemic index in pubertal aged young people who demonstrate relative insulin resistance suggesting that meals with a low glycaemic index may confer metabolic benefits in young people (102).

Given the evidence that sedentary lifestyles are a major contribution to cardiometabolic disease (103), there has been interest in the impact that promoting physical activity might have on cardiometabolic health. A 10 week programme of both high and medium intensity exercise has been shown to promote improvement in a range of cardiometabolic outcomes including blood pressure, body fat, and triglycerides in children ranging as young as 5 years old to adolescence (104, 105). Shorter duration interventions of only 7 and 5 weeks have also been found to be efficacious (106, 107), suggesting that programmed exercise may offer an effective intervention for reducing the risks of the metabolic syndrome in both an unselected population as well as in those with obesity (108, 109).

## Conclusions

There are many more observational studies evaluating evidence for factors which predispose to childhood obesity (and therefore for many, indirectly the metabolic syndrome) than those that evaluate influences on components of the metabolic syndrome. However, observational studies do not demonstrate cause and effect and a recent meta-analysis of the prevention and treatment of childhood and adolescent obesity reports discrepancies between observational and interventional studies (82). Although we have a reasonably good understanding of many of the predisposing causes of obesity, prevention strategies seem of limited value at present. At present, it is not entirely clear whether the major driver for childhood obesity is overconsumption of energy or decreasing physical activity (110) and so the primary target for interventions is not clear. Those interventions that are most likely to work, will target younger children in the hope of producing life-long changes to their behavior and that of their families. It is likely that complex multi-componented interventions will need to be developed, targeting both dietary energy intake and physical activity, to maximize impact (111), given the increasing adverse effects of our “obesogenic” environment. This will likely require interdisciplinary collaboration between public health and local government. By contrast, there are even fewer trials of effective interventions which specifically target components of the metabolic syndrome independent of effects on obesity and more research is required in this important area of public health.

## Author Contributions

JG has drafted this manuscript and takes responsibility for its content.

### Conflict of Interest

The author declares that the research was conducted in the absence of any commercial or financial relationships that could be construed as a potential conflict of interest.
